# Effect of Cage-Induced Stereotypies on Measures of Affective State and Recurrent Perseveration in CD-1 and C57BL/6 Mice

**DOI:** 10.1371/journal.pone.0153203

**Published:** 2016-05-04

**Authors:** Janja Novak, Jeremy D. Bailoo, Luca Melotti, Hanno Würbel

**Affiliations:** Division of Animal Welfare, VPH Institute, University of Bern, Länggassstrasse 120, 3012, Bern, Switzerland; Harvard University Faculty of Arts and Sciences, UNITED STATES

## Abstract

Stereotypies are abnormal repetitive behaviour patterns that are highly prevalent in laboratory mice and are thought to reflect impaired welfare. Thus, they are associated with impaired behavioural inhibition and may also reflect negative affective states. However, in mice the relationship between stereotypies and behavioural inhibition is inconclusive, and reliable measures of affective valence are lacking. Here we used an exploration based task to assess cognitive bias as a measure of affective valence and a two-choice guessing task to assess recurrent perseveration as a measure of impaired behavioural inhibition to test mice with different forms and expression levels of stereotypic behaviour. We trained 44 CD-1 and 40 C57BL/6 female mice to discriminate between positively and negatively cued arms in a radial maze and tested their responses to previously inaccessible ambiguous arms. In CD-1 mice (i) mice with higher stereotypy levels displayed a negative cognitive bias and this was influenced by the form of stereotypy performed, (ii) negative cognitive bias was evident in back-flipping mice, and (iii) no such effect was found in mice displaying bar-mouthing or cage-top twirling. In C57BL/6 mice neither route-tracing nor bar-mouthing was associated with cognitive bias, indicating that in this strain these stereotypies may not reflect negative affective states. Conversely, while we found no relation of stereotypy to recurrent perseveration in CD-1 mice, C57BL/6 mice with higher levels of route-tracing, but not bar-mouthing, made more repetitive responses in the guessing task. Our findings confirm previous research indicating that the implications of stereotypies for animal welfare may strongly depend on the species and strain of animal as well as on the form and expression level of the stereotypy. Furthermore, they indicate that variation in stereotypic behaviour may represent an important source of variation in many animal experiments.

## Introduction

Stereotypies are commonly defined as repetitive and invariant behaviour patterns without apparent goal or function [1,2]. They are prevalent in many captive species, including laboratory rodents [1–3]. Stereotypies are thought to reflect impaired welfare [2], as they usually develop in barren housing conditions [4–6]. Various (not necessarily mutually exclusive) mechanisms have been invoked to explain their development, including a lack of sensory and motor stimulation [2], chronic thwarting of highly motivated behaviour [2,3,7,8], attempts to cope with adverse environments [9], and central nervous system dysfunction [10,11]. However, attempts to link stereotypic behaviour with physiological or behavioural indicators of impaired welfare have produced mixed results. Jumping and bar-mouthing in laboratory mice, for example, have been suggested to develop from attempts to escape the cage [3,7,12]. Furthermore, bar-mouthing level has been found to correlate positively with corticosterone levels at weaning [13], a physiological measure of stress. However, in another study [7], no correlation with corticosterone levels was observed.

Most research so far has focused on behavioural and physiological measures of welfare. These are, however, difficult to interpret in terms of affective state, as they can be confounded by arousal and are therefore not reliable measures of affective valence (whether the animal is experiencing a positive or negative affective state) [14,15]. Studies in human psychology have shown that the valence of affective states influences cognitive processes, such as judgement, expectation, memory and attention [16–18]. For example, people in negative affective states tend to interpret ambiguous stimuli in a negative way, displaying a negative cognitive bias, while people in positive affective states display a positive cognitive bias [17,18].

Cognitive biases are sensitive to both short and long term changes in affect [16], and while various types of cognitive biases (judgement, memory and attention bias) have been investigated in humans, the most common type studied in non-human animals has been judgement bias (for review see [14]).

Judgement bias tasks have been implemented and validated across a number of animal species using short term experimental manipulations to alter affective state [19–22]. Based on this research, increasing evidence suggests that judgement bias can be used as a proxy measure of affective valence. However, the few studies employing judgement bias tasks to investigate the relation between the expression of stereotypic behaviour and affective states have produced conflicting results. For example, back-flipping in starlings has been associated with a negative cognitive bias [23]. In contrast, grizzly bears with higher levels of pacing displayed positive cognitive bias [24]. Furthermore, in capuchin monkeys only some forms of stereotypic behaviour (e.g., head twirls) were correlated with negative cognitive bias [22].

Similar inconsistent results have been found within species, such as in the common laboratory mouse, *Mus musculus*. While mice with higher overall stereotypy levels displayed a positive cognitive bias, this relation seems to depend on stereotypy form as, for example, no such relation was found with stereotypic bar-mouthing [25]. On the other hand, a spatial exploration task to assess judgement bias recently found that CD-1 mice with higher stereotypy levels displayed a negative judgement bias, but this result may have been confounded by the form of stereotypy performed [26].

Two possible reasons may explain these discrepancies between studies: differences in the types of tasks used and/or differences in the reinforcer value [14]. For example, in the exploration based task [26], a more aversive negative outcome (light on, white noise) was used compared to non-exploration based tasks using food-based positive and negative reinforcers [22–25], potentially increasing the likelihood of observing a more negative bias. However, conflicting results in non-exploration based studies [22–25] suggest that effects of stereotypy levels on cognitive biases may also depend on the form of stereotypy performed.

Studies relating the expression of stereotypic behaviour to measures of brain function also yielded conflicting results. Stereotypies have been related to impaired inhibitory control of behaviour [27,28], resulting in poor extinction learning [27,29–31], reversal learning [32,33], and other forms of perseverative responding [34,35]. These changes have been linked to an imbalance in the modulation of the direct and indirect pathways in the basal ganglia by dopamine [36], and may be a consequence of barren housing [37]. Current evidence indicates that stereotypies reflect a form of behavioural disinhibition termed “recurrent perseveration” [11,28,38]. Recurrent perseveration refers to the inappropriate repetition of a response to a stimulus [39,40], and positive correlations between stereotypy levels and recurrent perseveration have been found in a number of species, including blue tits [27], parrots [34], Malayan sun bears and Asiatic black bears [29,41], horses [30], bank voles [11], and mice [42]. Overall, animals with high levels of stereotypy show a strong tendency to repeat behavioural responses, and the fact that this relationship appears across a wide range of species implies a common underlying neural mechanism.

However, the evidence linking expression levels of stereotypic behaviour with perseverative responding is ambiguous. In laboratory mice for example, overall stereotypy level was positively correlated with measures of recurrent perseveration in C57BL/6 [42], but not CD-1 mice [4,5,43]. Similarly, studies in birds found that route-tracing and oral stereotypies in songbirds [27] and parrots [34], but not back-flipping and route-tracing in starlings [44], reflect recurrent perseveration. Furthermore, in mink [35,45], deer mice [32] and non-human primates [31,33] only some stereotypies, but not others, were found to correlate positively with recurrent perseveration. Such inconsistencies could be due to different tasks used in the measurement of recurrent perseveration. Many studies have used a two-choice guessing task, which requires a simple response to a stimulus [5,27,35,42,44], while other studies have implemented extinction learning [4,27,31,43], which may be affected by other processes (e.g., learning, stuck-in-set perseveration [11,27]). However, positive correlations have been found using both of the above mentioned tasks, which indicates that the relation (or lack thereof) between stereotypy and perseveration may depend on the form of stereotypy.

Animals reared under barren housing conditions tend to display elevated levels of perseverative behaviour [4,32,45] (but see [43] and [35]) compared to animals reared under enriched conditions. Similarly, wild caught striped mice are less perseverative than captive reared mice [46], supporting the hypothesis that higher levels of perseveration may be linked to poor laboratory housing conditions. However, few studies have investigated the relation between perseverative behaviour and affective states. Impaired decision making and impaired behavioural control are sources of frustration in humans [39,47] and could possibly be sources of frustration in animals as well. For example, the level of head twirling in non-human primates was not only correlated with recurrent perseveration [31,33] but was also linked to negative cognitive bias [22]. Conversely, while back-flipping starlings displayed a negative cognitive bias [23], this stereotypy was not associated with recurrent perseveration [44]. Similarly, studies comparing recurrent perseveration with indicators of frustration (motivation to gain access to enrichments and corticosterone levels) in CD-1 mice found no link between these two measures [43].

Taken together, current evidence linking the expression of stereotypic behaviour to the valence of affective states is ambiguous. The same is true for evidence linking the expression of stereotypies to measures of impaired behavioural control. These inconsistencies may at least in part be explained by different forms of stereotypy, which may involve different motivational factors relating to different underlying mechanisms and may thus also have different welfare implications. Several authors have recognised the importance of differentiating between different stereotypy forms when examining the underlying mechanisms [2,11,31,32,45], and as discussed above, different conclusions have been reached when different forms of stereotypy were considered.

Most studies in rodents have described stereotypies as a homogenous group of abnormal behaviours when exploring their relation with measures of impaired behavioural inhibition and affective state [4,5,26,42,43]. In the present study we therefore evaluated in two strains of mice, the relation between the form and level of stereotypic behaviour to variation in measures of cognitive bias and recurrent perseveration. To measure cognitive bias, we used an exploration based cognitive bias task, previously used in rats [48] and mice [26]. Mice were trained on a spatial discrimination task, where two arms in a radial maze predicted a positive outcome, and the two opposite arms predicted a negative outcome. After the training session, mice were given access to the previously unavailable intermediate arms. We hypothesised, that if some stereotypies reflect negative affective states, mice with higher levels of those stereotypies should display a more negative cognitive bias by avoiding ambiguous arms. To measure recurrent perseveration, the mice were tested in a two-choice guessing task [34]. Similarly, we hypothesized that if some stereotypies reflect recurrent perseveration, mice with higher levels of those stereotypies should generate a more perseverative pattern of responding.

## Material and Methods

### Animals and husbandry

80 female CD-1 and 80 female C57BL/6 mice were purchased in two replicates (40 mice of each strain) two months apart from Harlan Laboratories (Netherlands), at three weeks of age. They were randomly assigned to Type II cages (22 x 16 x 14 cm, Techniplast) in pairs, with wood chips (Lignocel select, Rettenmaier & Söhne GmbH, Germany) as bedding but no nesting material, as nesting material considerably attenuates stereotypic behaviour [6,49,50]. Food (Kliba Nafag #3430, Provimi Kliba AG, Switzerland) and water were provided *ad libitum*, and animals were kept on a reversed 12:12 hour dark:light cycle, with lights off at 09:00 h.

### Experimental design

Previous studies using different tasks found that stereotypy level was positively correlated with measures of recurrent perseveration in C57BL/6 mice [42] but not in CD-1 mice [5,43], therefore these two strains were used in the present study. For unknown reasons, one CD-1 and one C57BL/6 mouse died before the onset of data recording, so those cages were excluded from the experiment. The remaining 154 mice were screened for the expression of different forms of stereotypic behaviour at week 26 of age. All cages were recorded for two consecutive days and videos were screened using continuous observations to assess the form (but not level) of stereotypic behaviour performed, based on our previously validated ethogram [25] (Table 1).

**Table 1:** Ethogram for the recording of home cage behaviour.

Category	Name	Definition
**General activity**	Inactive	Sitting or lying motionless throughout the 15 s interval, occasionally interrupted by brief single twitches lasting no longer than 5s.
	Active	All activities except the stereotypic activities listed below.
**Stereotypic behaviour**	Bar-mouthing	Chewing on a bar with the bar held in the gap between incisors and molars (diastema) while hanging on the cage lid (with all four paws or the forepaws only) or standing on the hind legs. Bar-mouthing may be performed on the spot or by moving along the bar while chewing.
	Circling	Running around the cage in circles.
	Cage-top twirling	Spinning around the longitudinal body axis while hanging on the cage lid with the forepaws.
	Back-flipping	Backward flip from one cage wall or the food rack towards the opposite cage wall, with or without touching the cage lid and/or the opposite cage wall during the flip.
	Route-tracing on the cage-lid	Moving along the same route on the cage lid with all four legs.

Behaviour patterns were considered as stereotypic if the same movement sequence was repeated continuously for at least 3 s (bar-mouthing) or at least three times in a row without pauses longer than 3 s between bouts (cage-top twirling, back-flipping, route-tracing, circling).

**Fig 1:**
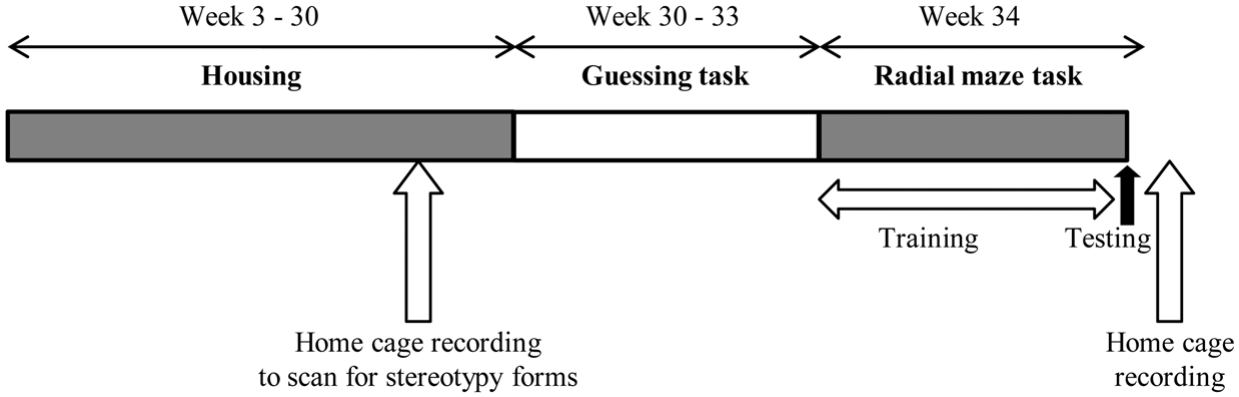
Timeline of the study.

Based on the stereotypy forms observed at the screening phase, 60 mice per strain were chosen for testing, so that all stereotypy forms observed were included in the sample at the onset of testing. Mice were tested for recurrent perseveration in a two-choice guessing task from week 30–33 of age, followed by a cognitive bias task on a radial arm maze at 34 weeks of age. After testing, home cage behaviour was recorded again (at 35 weeks of age), to assess individual levels of expression of the different forms of stereotypic behaviour (Fig 1).

The number of mice displaying each stereotypy form recorded at screening and after testing are listed in S1 Table. Since the number of mice performing cage-top twirling with bar-mouthing (CT-BM) and back-flipping (BF) at the time of screening were low, all individuals performing those stereotypies were chosen for testing. In mice performing no stereotypy (NS) and bar-mouthing (BM), 18 and 20 mice, respectively, were chosen randomly (using a computer generated random sequence). Mice remained housed with the same cage-mates throughout the study. Therefore, in some cases both mice from the same cage were tested and in other cases only one mouse from the cage was tested.

Some mice which displayed BM or CT-BM during the screening phase, displayed NS at the time of testing and vice versa (which only became apparent after testing, at the time of stereotypy recording), resulting in an unequal number of mice in those two groups. In C57BL/6 mice, 20 mice from NS, route-tracing (RT) and route-tracing with bar-mouthing (RT-BM) were chosen randomly for testing, but most mice performed RT-BM at the time of testing.

### Home cage behavioural observations

Home cage behaviour was recorded using IR cameras (VC Videocomponents GmbH, Germany). For individual recognition, one mouse per cage was marked one day before the start of home cage recording, using a permanent marker (Edding 500) while the cage-mate was sham marked. From both days of video recording, the mice were observed for the first 15 min of the 2^nd^, 3^rd^, 4^th^ and 5^th^ hour of the dark phase. Behaviour was sampled using one-zero sampling with 15 s intervals [5,43,51], yielding 480 data points per mouse across the two observation days. The ethogram used for behavioural recording is presented in Table 1. The level of each form of stereotypic behaviour was assessed as a proportion of active time (where active time was calculated as proportion of observed time).

### Guessing task

**Fig 2:**
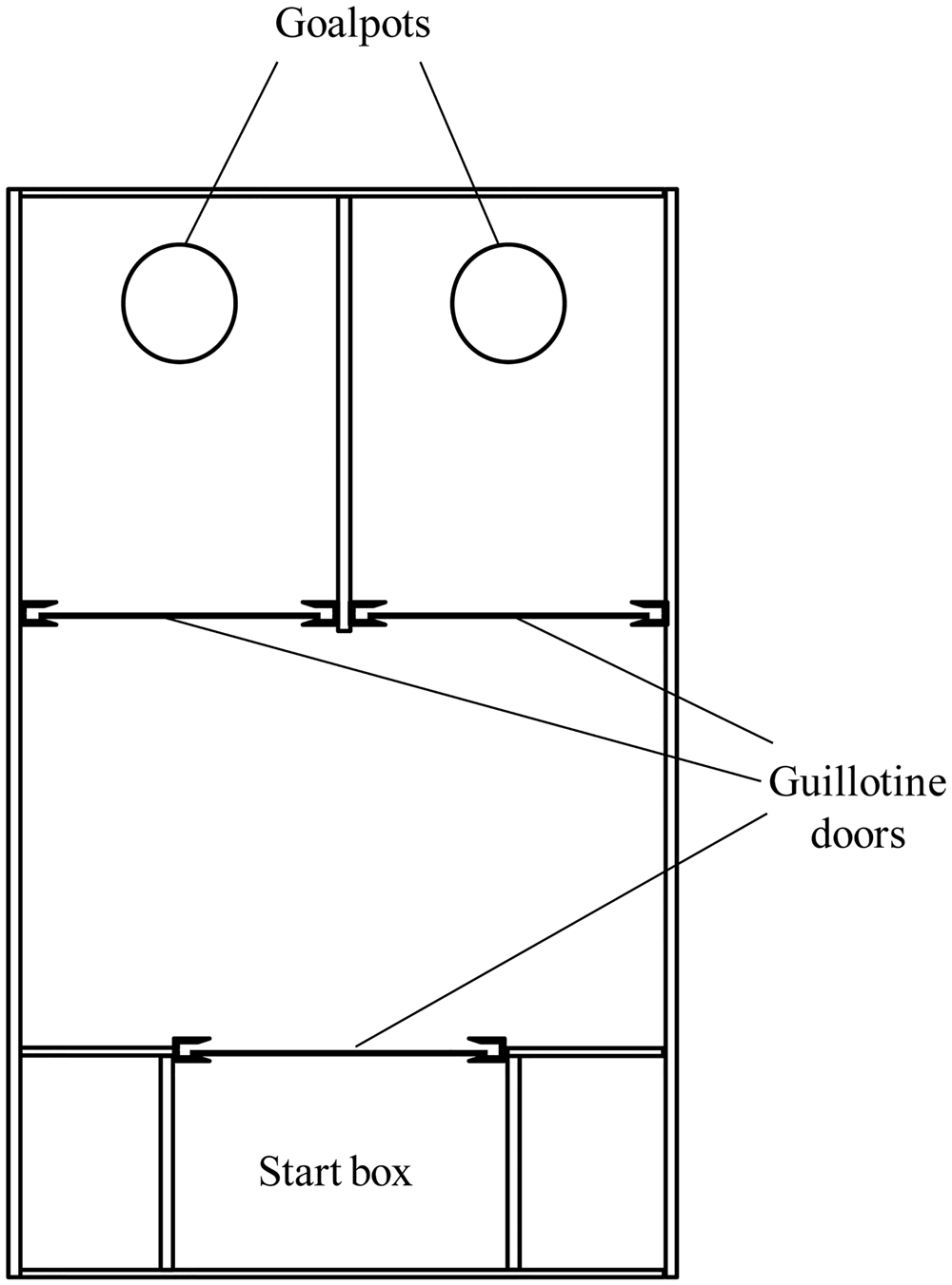
Apparatus used for the guessing task. Both compartments and the start box were separated by guillotine doors operated manually.

We used the same experimental protocol as described by Garner et al. [42] and Gross et al. [4] to measure recurrent perseveration. In contrast to the above studies, which used 100 response sequences, we only used 80, as previous pilot studies confirmed that this was sufficient for analysis. A previous study found that CD-1 mice tended to display high rates of spontaneous alternation (LRLR and RLRL) when tested on a guessing task in a T-maze [4], possibly due to a natural tendency of mice to spontaneously alternate in spatially oriented tasks [52,53]. To avoid a possible confound of alternation on measures of perseveration, we used an apparatus with two adjacent goal compartments (Fig 2) that has been shown to eliminate spontaneous alternation in both CD-1 [25] and C57BL/6 strains [42].

#### Apparatus

The apparatus consisted of a box made of black plastic measuring 20 x 50 cm (height: 15 cm), which contained a start box (10 x 10 cm) and two goal compartments (10 x 20 cm), each containing a goalpot (Fig 2).

#### Test procedure

Mice were food restricted for the duration of the task. Starting three days before the onset of testing, mice were fed a reduced amount of food once a day (3–4 g of food per day/per cage). Subjects were weighed daily to ensure that their body weight was maintained at about 90% of their body weight when fed *ad libitum*.The task was conducted under red light, between 10:00 h and 14:00 h. The test order of cages was randomized daily using a computer generated random sequence, and the two mice from the same cage (where applicable) were tested at the same time and in the same room, each by one experimenter. If a mouse did not perform the task, it was put back in the home cage and tested at the end of the session. In case an animal’s weight dropped below 85%, it was put in a separate cage and fed *ad libitum* for 30 minutes. Rewards used in the task were 20 mg chocolate flavoured pellets (Dustless Precision Pellets, Bio-Serv^™^). In all trials, both goalpots contained an inaccessible pellet at the bottom which was covered with wire mesh and served as control for odour cues. Between mice, but not between trials, the apparatus was cleaned with a 70% ethanol solution.

**Habituation to reward:** One week prior to testing, mice were given chocolate flavoured pellets in their home cage daily (four pellets per cage), to reduce neophobia and to habituate them to the food reward.

**Habituation to apparatus:** On day one, each mouse was placed in the apparatus for ten minutes in a pre-specified random order. Both goalpots were present and both contained one chocolate pellet. Chocolate pellets were also scattered throughout the apparatus (except in the two goal compartments).

**Shaping:** On day two each mouse received 12 training trials in which both goalpots were baited. As soon as the mouse entered one compartment, access to the other compartment was blocked by closing the guillotine door. If the mouse chose the same side three times in succession, that side was closed in the following trial to avoid shaping the mouse to one side. A trial was completed when the animal’s head (nose) was above the goalpot, after which the animal was left to eat the reward. The mouse was then returned to the start box by the experimenter and the next trial begun.

**Testing:** The test phase consisted of 80 trials conducted over a maximum of three sessions. For each trial, the start box door was opened and once the animal had made a choice, the other compartment was closed. The animal was left to eat the pellet and then returned to the start box. Each session was terminated after 30 minutes or as soon as the mouse started showing off-task behaviour (cf., [11]). On each trial, only one compartment was baited, with a probability equalling the proportion of responses to the other side in the previous twenty trials. In trials 1 to 19, the side bias was calculated from all previous trials (side bias was measured as probability of choosing the right goalpot). This randomization procedure was used to eliminate side biases which may confound the experimental paradigm and was determined by a custom written computer program [27]. Although reward side is unpredictable, choosing each side equally often will maximize the number of rewards. The mouse can do so by producing either a random or patterned sequence of responses. Patterned sequences (which show high sequential dependence), can be apparent as either a series of repetitions or alternations, and indicate recurrent perseveration [38,42,54].

#### Outcome measures

Perseveration score (logit[P]) was used as the primary outcome measure of recurrent perseveration. The score is calculated using 3^rd^ order Markov chain analysis [34], which describes the probability of a behaviour occurring as a function of previous behaviour (where the 3^rd^ order considers the three previous behavioural responses) and provide a way to assess sequential independence. These analyses were performed by a custom written computer program which calculated the observed and expected probabilities of each choice. Then the sum chi-square was calculated from the observed and expected values. The probability of each sum chi-square (p) indicates the probability of sequential independence of the observed sequence. Therefore, recurrent perseveration was calculated by (1−p), where 1 represents a completely perseverant sequence and the data were logit transformed (logit[P]).

Numbers of pure repetitions (RRRR, LLLL) and pure alternations (RLRL, LRLR) were considered as secondary outcome measures of a non-random search strategy. Distribution of tetragrams (sequences of four trials) was examined by dividing each response sequence consisting of 80 trials per mouse into 77 overlapping tetragrams. 16 configurations of tetragrams were possible, of which two were pure repetitions and two were pure alternations. A random search strategy would be characterized by an equal distribution of all possible configurations (77/16 = 4.8), whereas perseverative behaviour should result in sequences characterized by higher rates of alternations or repetitions [4]. Thus, the frequencies of repetitions and alternations were counted for each subject and compared between mice with different forms and levels of stereotypy.

### Cognitive bias task

#### Apparatus

**Fig 3:**
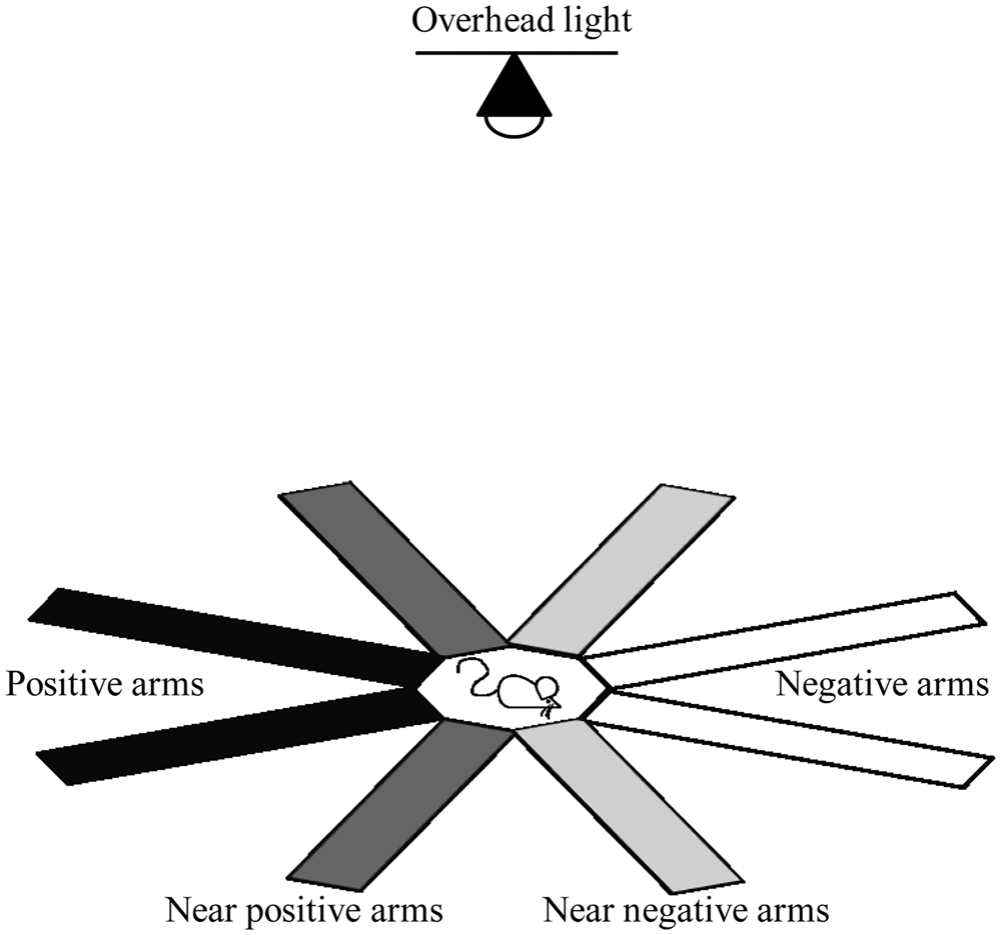
Eight arm radial maze used for testing cognitive bias. The radial maze contained four reference arms, two associated with positive outcomes (black) and two with negative outcomes (white), and four ambiguous arms, two of them adjacent to positive arms (dark grey) and the other two adjacent to negative arms (light grey). During training the four ambiguous arms were closed, while during testing all eight arms were open. The arms of the maze were not coloured. We highlighted them in the figure to make it easier for the reader to distinguish between positive, near-positive, near-negative and negative arms.

The cognitive bias task was implemented using an eight arm radial maze (Med-Associates Inc.; Fig 3). Each arm was 46 cm long and 9 cm wide and the central arena was 28 cm in diameter. The bottom of the maze was backlit with infrared light which eliminated tracking errors associated with automated tracking [55]. A computer with Ethovision XT software (Noldus, Version 9) recorded the animal’s movement in the maze via a video camera equipped with an infrared pass filter, and automatically activated contingencies when the animal entered an arm or the end of an arm. The detection settings for Ethovision XT were selected so that both the percentage of samples in which the subject was not found and the percentage of samples skipped were less than 1% per trial. For both training and testing, the time spent in each arm and the number of arm entries was automatically recorded.

#### Test procedure

**Training:** Mice were trained for one ten minute session each day for five consecutive days starting at 10:00 h. The order of cages was randomised daily and the order of training of the two mice per cage was reversed on alternate days. During training, only four reference arms—two pairs of adjacent arms opposite to each other—were open; two positive arms and two negative arms (Fig 3). The remaining four arms were closed during the training sessions. Each session started with the overhead light on (400 lux). Reaching the ends of the positive arms turned the overhead light off either until the mouse entered a negative arm or until it exited a positive arm and stayed in the central arena for 20 s. Reaching the end of the positive arms also activated a pellet dispenser, dispensing a 20 mg chocolate flavoured pellet (the same type as used in the guessing task). When entering negative arms, the overhead light came on and stayed on until the animal entered the end of a positive arm. Entering the end of negative arms also triggered a burst of white noise which remained on until the animal exited the negative arm.

**Testing:** After five days of training, the mice were tested for responses to ambiguous arms. The test session was identical to the training sessions, with the exception that all eight arms were now available for exploration. Contingencies for reference arms remained the same as during training, while entering and reaching the ends of the ambiguous arms activated no contingencies. Both during training and testing, number of arm entries and time spent in each arm were automatically recorded by Ethovision XT.

#### Outcome measures

Time spent in arms is presented as relative to trial duration, and number of arms entered as relative to number of all arm entries. Since time spent in arms and number of arm entries was positively correlated for all arms, time spent in arms was used as the primary outcome measure of arm preference in the analysis. When an effect of stereotypy on time spent in arms was observed, we additionally looked at the number of arm entries. For the comparison of time spent in positive and negative reference arms, we calculated a “positive arm score” by dividing the difference between the time spent in positive arms minus time spent in negative arms by the time spent in all reference arms. To compare visits to reference arms and ambiguous arms, we calculated a “reference arm score” by calculating the difference between the time spent in reference arms minus the time spent in ambiguous arms, divided by the time spent in all arms. Furthermore, we calculated an “ambiguous arm score” by calculating the difference in times spent in near positive arms and near negative arms divided by the time spent in all ambiguous arms. A higher reference score simply indicates that animals spent more time in reference arms and less time in ambiguous arms and does not allow for clear interpretation whether this difference was due to preference for reference arms or active avoidance of ambiguous arms. Therefore, our use of the term “avoidance” throughout the manuscript is equivalent, yet complementary, to the term preference. Number of all arms entered was used as a measure of activity and overall exploration in the radial maze.

### Ethical statement

This study was carried out in strict accordance with the recommendations in the Animal Welfare Ordinance (TSchV 455.1) of the Swiss Federal Food Safety and Veterinary Office. It was approved by the Cantonal Veterinary Office in Bern, Switzerland (Permit Number: BE12/12).

### Statistical analyses

All statistical analyses were performed with R (version 2.15.3) and R Studio (version 0.98.507). The function lmer in the R package “lmer4” and “lmerTest” was used to fit linear mixed effects models [56] and *P*-values below 0.05 were considered significant for all analyses. The assumptions of normally distributed errors and homogeneity of variance were examined graphically with the use of the Normal plot and the Tukey-Anscombe plot. To satisfy these assumptions, level of stereotypic behaviour was square-root transformed. Stereotypy level is reported as proportion of active time. Results shown are untransformed means ± SEM. Data for each strain were analysed separately.

For the radial maze data, training and test data were analysed for each of the previously mentioned outcome variables. For test data, stereotypy form, stereotypy level, and the interaction between stereotypy form and level were included in the model as predictors with individual nested within cage as a random effect. Additionally, for training data in the radial arm maze, session and the interaction between session and stereotypy form, stereotypy level and stereotypy form x level were included as predictors in the model. In all analyses non-significant predictors were excluded stepwise (starting with interactions then non-significant main effects) to produce a final model. Bonferroni corrected *post hoc* tests were used to probe significant main effects and interactions.

## Results

### Missing data

For unknown reasons, two C57BL/6 mice died in the course of the study. In the guessing task, three CD-1 and six C57BL/6 mice showed off task behaviour in the shaping period and did not complete the 12 shaping trials. They were excluded from the analysis. Furthermore, due to a technical failure, guessing task data from eight CD-1 and eight C57BL/6 mice were lost. In the cognitive bias task, four CD-1 mice performed circling behaviour in the radial maze and never performed the task. Mice with missing data for either test were excluded from the analysis. Finally, two C57BL/6 mice were excluded after recording the expression of stereotypic behaviour as they only performed RT compared to all other mice which performed RT-BM, resulting in a final sample of 44 CD-1 mice and 40 C57BL/6 mice.

### Expression of stereotypic behaviour

**Fig 4:**
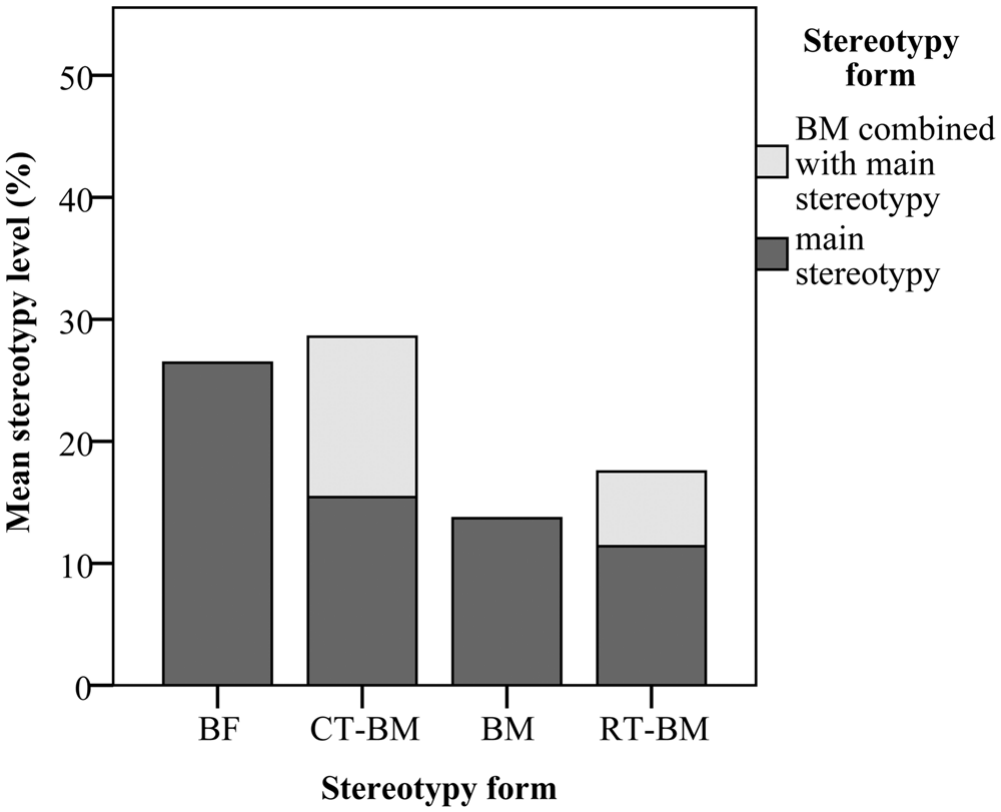
Level of stereotypic behaviour by stereotypy form. In groups of mice performing two forms of stereotypies, dark grey bars represent the level of the main stereotypy form performed (CT or RT) and light grey bars represent the level of BM performed. Overall level of stereotypy in CT-BM and RT-BM mice were lower as shown here, as in some observation intervals, mice performed both forms of stereotypy. Therefore, the levels of the two stereotypies were not additive. BF = back-flipping, CT-BM = cage-top twirling and bar-mouthing, BM = bar-mouthing, RT-BM = route-tracing and bar-mouthing.

Of the 44 CD-1 mice, eight mice performed NS, 20 mice performed BM, nine mice performed BF, and seven mice performed both CT and BM (CT-BM) (Fig 4). Among these mice, levels of CT and BM were not correlated (*r* = 0.10, *P* > 0.05, *df* = 6, controlling for cage and replicate). To analyse the effect of form of stereotypy on outcome measures, CD-1 mice were therefore split into the four groups NS, BM, BF, and CT-BM. Total level of stereotypy was affected by the form of stereotypy (*F*_*(2*,*41)*_ = 4.52, *P* < 0.05), with levels of BM being significantly lower than levels of BF. All 40 C57BL/6 mice performed both RT and BM (RT-BM) (Fig 4). Levels of RT and BM were positively correlated (*r* = 0.52, *P* < 0.05, *df* = 39, controlling for cage and replicate).

### Guessing task

**Fig 5:**
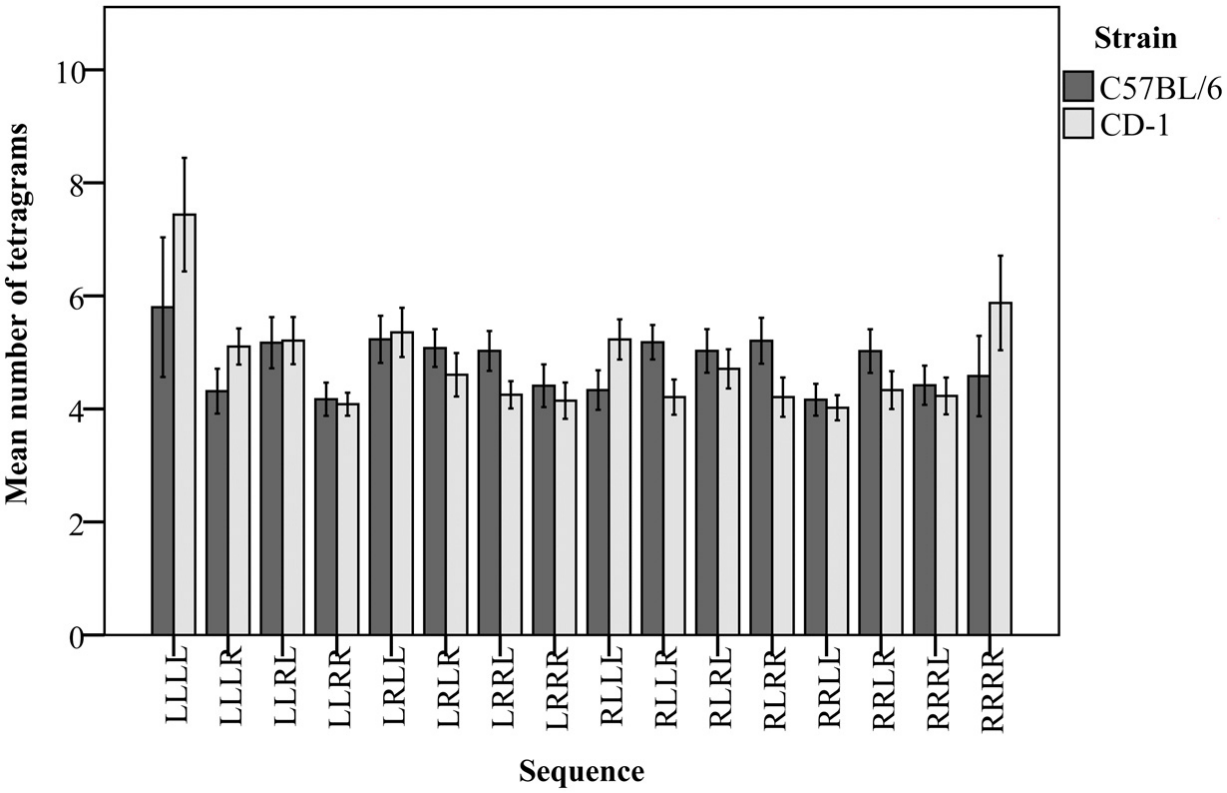
Distribution of tetragrams of left (L) and right (R) choices in the guessing task.

The frequencies of the 16 different tetragrams of left (L) and right (R) choices were not equally distributed in both CD-1 (*F*_*(15*,*542)*_ = 8.68, *P* < 0.05) and C57BL/6 mice (*F*_*(15*,*531)*_ = 2.40, *P* < 0.05; Fig 5).

#### Effect of stereotypy expression on measures of recurrent perseveration

Measures of recurrent perseveration for the two strains of mice are listed in S2 Table, and the effects of form and level of stereotypy expression on perseveration measures are listed in Table 2.

**Table 2:** Effect of stereotypy level on measures of recurrent perseveration in the guessing task.

	CD-1			C57BL/6
	level x form	level	form	level
	*F*_*3*,*41*_	*F*_*1*,*43*_	*F*_*3*,*41*_	*F*_*1*,*39*_
perseveration score	0.99	1.42	0.47	0.22
repetitions	0.92	1.20	1.35	7.78*
alternations	0.87	1.26	0.60	1.84
*p* making correct choice	0.61	1.12	0.82	0.33
*p* displaying side bias	0.07	0.002	2.95*	0.47

* P < 0.05

In CD-1 mice, we found no effect of stereotypy level or form on the primary or secondary perseveration measures. Stereotypy form affected the probability of displaying a side bias (*F*_*(3*,*41)*_ = 2.95, *P* < 0.05), with NS and BM mice displaying stronger side biases (NS displaying 58 ± 4% and BM; displaying 43 ± 3% choices to the right side), compared to CT-BM (48 ± 4%) or BF mice (48 ± 3%).

**Fig 6:**
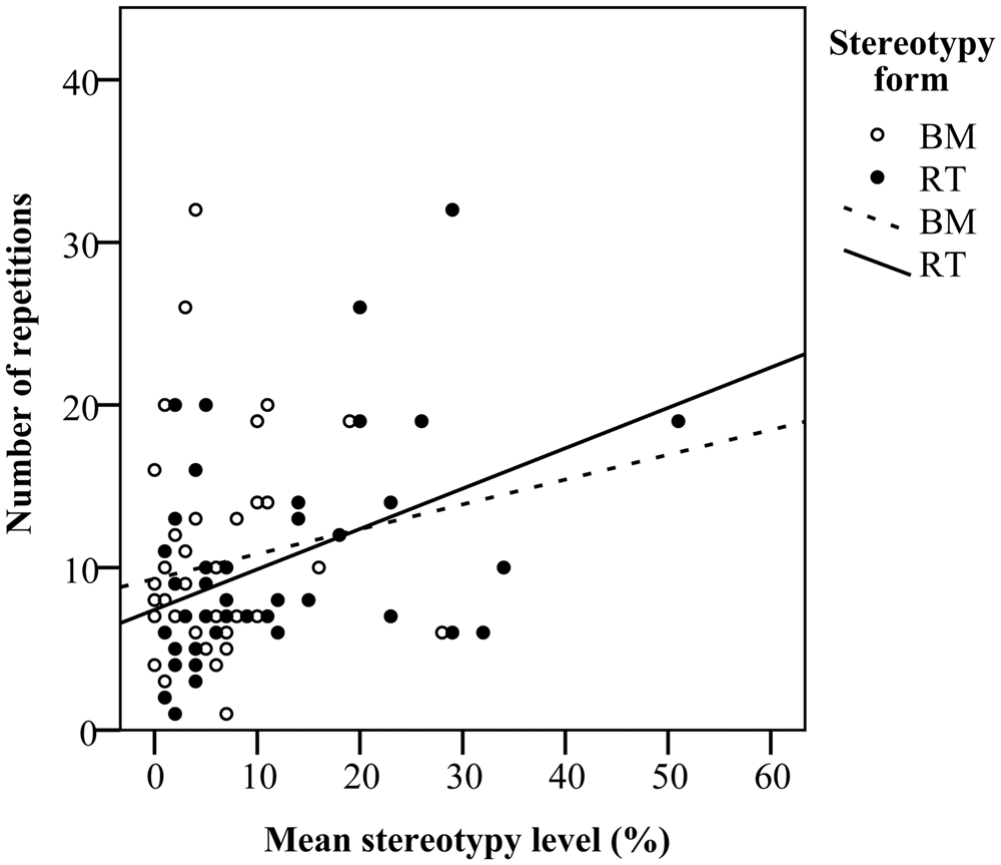
C57BL/6 mice with higher levels of RT displayed higher numbers of pure repetitions (LLLL, RRRR) in the guessing task. BM level did not correlate with number of repetitions.

Similarly, in C57BL/6 mice we found no effect of total stereotypy level on the perseveration score and the number of alternations, however, mice with higher levels of stereotypy made more pure repetitions (*F*_*(1*,*39)*_ = 7.78, *P* < 0.05). This effect was associated with the level of RT (*F*_*(1*,*39)*_ = 9.13, *P* < 0.05), but not BM (*F*_*(1*,*39)*_ = 0.91, *P* > 0.05; Fig 6). C57BL/6 mice with higher levels of repetitions also made fewer correct choices, indicating a suboptimal search strategy (*F*_*(1*,*39)*_ = 45.71, *P* < 0.05).

### Cognitive bias task

All measures of exploration from the training and test sessions are listed in S3 Table. In the CD-1 strain, mice spent more time in positive arms compared to negative arms, regardless of the training session (*t(219)* = 15.23, *P* < 0.05). Similarly, C57BL/6 mice spent more time in the positive arms compared to the negative arms (*t*(199) = 77.53, *P* < 0.05), a difference that was observed regardless of session. During the test session, both strains discriminated between positive and negative reference arms, spending more time in positive arms (CD-1; *t*(*43*) = 5.88, *P* < 0.05 and C57BL/6; *t*(*39*) = 2.89, *P* < 0.05). Both strains also spent more time in near positive compared to near negative arms (CD-1: *t*(*43*) = 3.24, *P* < 0.05; and C57BL/6 (*t*(*39*) = 2.45, *P* < 0.05).

#### Effect of stereotypy expression on measures of cognitive bias

The effects of overall stereotypy level, form and training session on measures of cognitive bias task are listed in Tables 3 and 4.

**Table 3:** Effects of stereotypy level, form and session on the cognitive bias task measures in CD-1 mice during training and testing.

	group of mice based on stereotypy form performed					
	**level x form x session**	**level x session**	**form x session**	**level**	**form**	**session**
	***F***_***12*,*172***_	***F***_***4*,*172***_	***F***_***12*,*172***_	***F***_***1*,*43***_	***F***_***3*,*43***_	***F***_***4*,*215***_
**training**						
positive arm score	0.93	2.34	0.93	7.87*	0.30	0.98
number of arms entered	1.57	5.18*	1.51	2.13	8.41*	5.68*
	**level x form**	**level**	**session**			
	***F***_***3*,*41***_	***F***_***1*,*43***_	***F***_***3*,*41***_			
**testing**						
positive arm score	0.32	0.36	0.15			
reference arm score	3.59*	0.17	1.24			
ambiguous arm score	1.56	0.08	0.20			
number of reference arms entered	1.03	0.11	2.41			
number of ambiguous arms entered	0.89	0.11	2.39			
number of arms entered	5.92*	0.02	0.13			

* P < 0.05

**Table 4:** Effects of stereotypy level, form and session on the cognitive bias task measures in C57BL/6 mice during training and testing.

	group of mice based on stereotypy form performed		
	**level x session**	**level**	**session**
	***F***_***4*,*156***_	***F***_***1*,*39***_	***F***_***4*,*39***_
**training**			
positive arm score	0.41	11.59*	1.50
number of arms entered	1.29	22.96*	17.88*
	**level**		
	***F***_***1*,*39***_		
**testing**			
positive arm score	2.63		
reference arm score	1.40		
ambiguous arm score	0.01		
number of arms entered	0.89		

* P < 0.05

**Fig 7:**
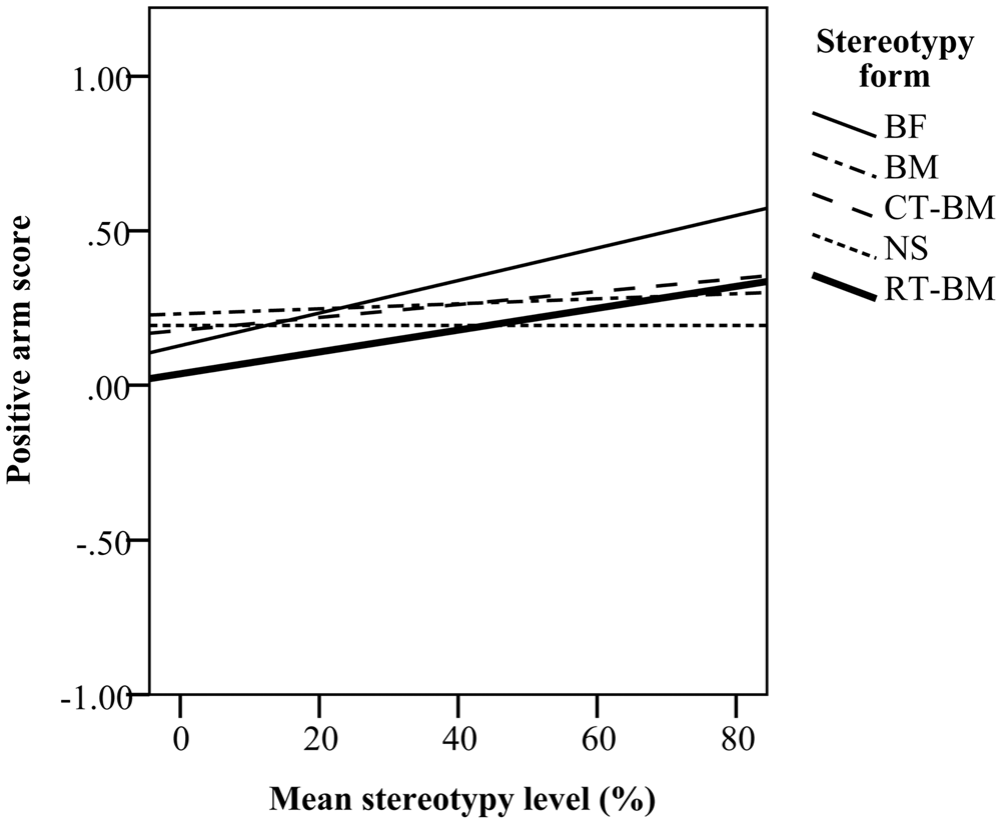
Mice with higher levels of stereotypy spent more time in positive arms during training. A positive arm score of 0 indicates that mice spent the same amount of time in positive as in negative arms. An increasing positive arm score (from 0 to 1) indicates a higher preference for positive arms, while a decreasing score (from 0 to -1) indicates a lower preference for positive arms.

**Fig 8:**
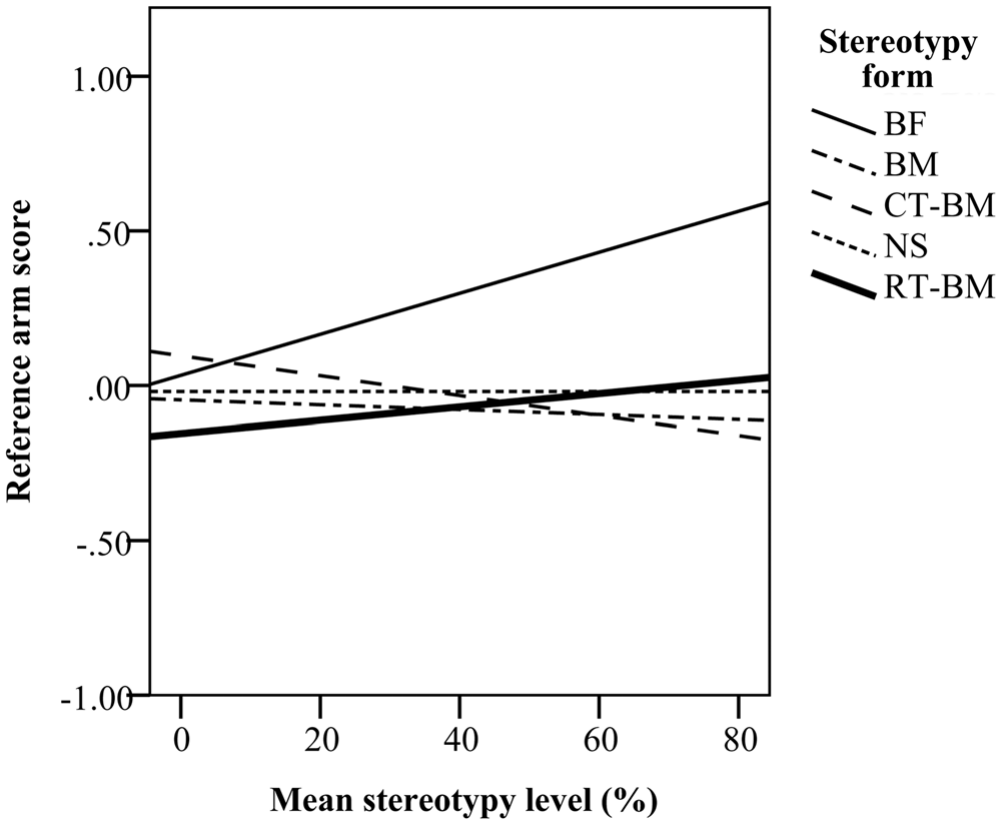
Time spent in reference and ambiguous arms during testing. Higher reference arm score indicates preference for reference arms and avoidance of ambiguous arms, while a negative score indicates more time spent in ambiguous arms.

In the CD-1 strain, mice with higher levels of stereotypy showed a stronger preference for positive arms during training (*F*_*(1*,*43)*_ = 7.87, *P* < 0.05; Fig 7). During testing when all eight arms were open, stereotypy level also affected exploration, however, this effect depended on the form of stereotypy performed (*F*_*(3*,*41)*_ = 3.59, *P* < 0.05). BF mice increased their time in reference arms and avoided the ambiguous arms as the level of stereotypy increased (*β* = 66.75), spending up to 75% of their time in the reference arms. CT-BM and BM mice decreased their time in reference arms and spent more time in ambiguous arms as stereotypy level increased (*β* = -38.00 and *β* = -7.88, respectively; Fig 8). However, both CT-BM and BM mice still spent almost half of their time in the reference arms and the other half in the ambiguous arms. This effect of time spent in arms was evident despite no difference in number of arm entries to either reference or ambiguous arms (Table 3).

Similar to CD-1 mice, C57BL/6 mice with higher levels of stereotypy spent more time in positive arms relative to negative arms in the training (*F*_*(1*,*39)*_ = 11.59, *P* < 0.05, Fig 7). This preference was associated with the level of RT (*F*_*(1*,*39)*_ = 6.45, *P* < 0.05) but not BM (*F*_*(1*,*39)*_ = 0.10, *P* > 0.05). In C57BL/6 mice, stereotypy level had no effect on exploration of ambiguous arms during testing (Fig 8).

**Fig 9:**
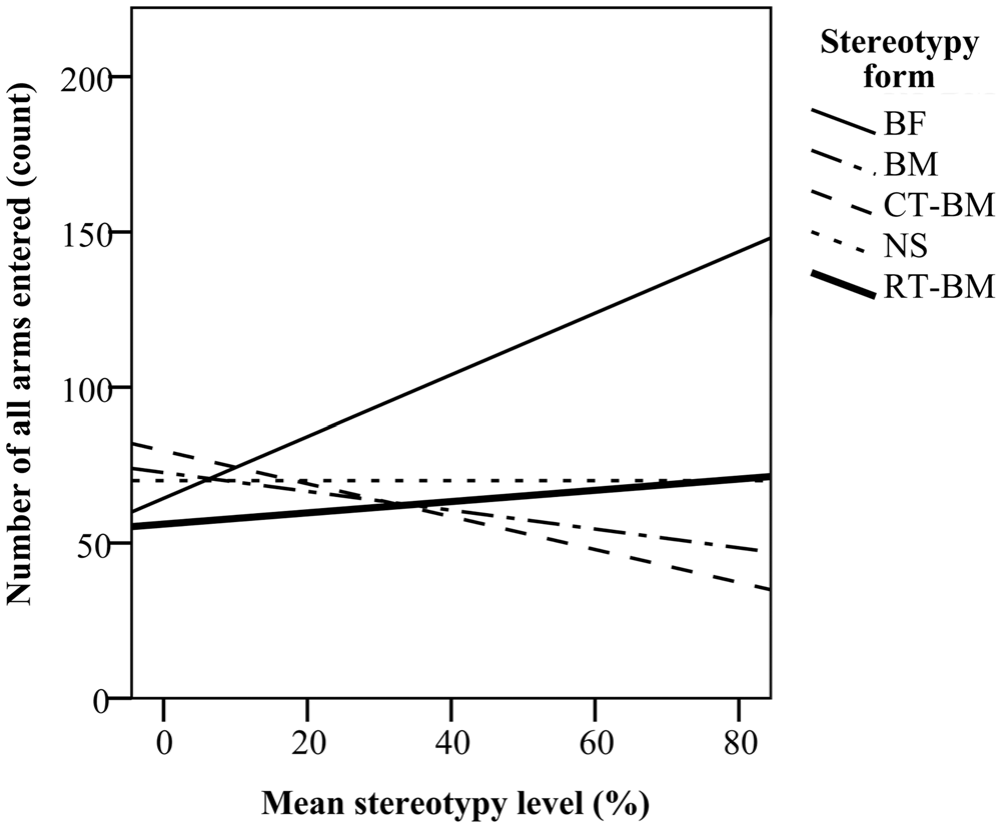
Number of all arms entered during testing in relation to the level of stereotypic behaviour for each stereotypy form.

The effect of stereotypy level on activity measures depended on the level and form of stereotypy performed. CD-1 mice decreased their activity across the training sessions from 61 ± 2 arms entered in the first session, to 55 ± 3 in the last session. However, this decrease was greater for mice with lower levels of stereotypy (*F*_*(4*,*172)*_ = 5.18, *P* < 0.05). BF mice were also more active during training (*F*_*(3*,*41)*_ = 8.41, *P* < 0.05), entering on average 74 ± 4 arms, compared to NS (53 ± 2 arms), BM (53 ± 1 arms) and CT-BM mice (51 ± 2 arms). During testing, a significant interaction between stereotypy level and form was observed, where mice with higher levels of BF were more active and entered more arms (*β* = 129.42), while CT-BM and BM mice with higher levels of stereotypy entered fewer arms (*β* = −20.66 and *β* = -30.30, respectively, Fig 9).

C57BL/6 mice also decreased activity across training sessions, from 61 ± 1 arms entered in the first session to 48 ± 2 arms entered in the last session (*F*_*(4*,*39)*_ = 17.88, *P* < 0.05). Additionally, mice with higher stereotypy levels were more active (*F*_*(1*,*39)*_ = 22.96, *P* < 0.05) and this effect was associated with the level of RT (*F*_*(1*,*39)*_ = 6.51, *P* < 0.05), but not BM (*F*_*(1*,*39)*_ = 2.85, *P* > 0.05). There was no effect of stereotypy level on radial maze exploration in the test phase.

## Discussion

The main aim of this study was to explore the effects of cage-induced stereotypies in mice on measures of affective state and recurrent perseveration, and to assess how these effects vary depending on the specific form and expression level of stereotypic behaviour. Overall stereotypy level affected exploration in the cognitive bias test in CD-1 mice, and this effect was influenced by the form of stereotypy performed. With increasing levels of stereotypic behaviour, CD-1 mice displaying BF showed increasing avoidance of ambiguous arms, indicating a more negative cognitive bias. No such effect was observed in CD-1 mice displaying BM or CT-BM or in C57BL/6 mice with RT-BM. Furthermore, stereotypy level was not correlated with perseveration score in either strain; however, in C57BL/6 mice, the level of RT, but not BM, was positively correlated with the number of pure repetitions in the guessing task, a secondary measure of recurrent perseveration.

### Expression of stereotypic behaviour

In C57BL/6 mice, stereotypic behaviour was more prevalent as only three non-stereotypic mice were observed in this strain compared to 14 in the CD-1 strain. The prevalence of forms of stereotypies differed across the two strains. In the C57BL/6 strain almost all mice performed both RT and BM combined, while there was greater intra-strain variability in the forms of stereotypy performed in CD-1 mice. The different forms of stereotypies observed are consistent with what other studies have reported [4,5,57,58], but stereotypy levels were slightly lower (15.34 ± 2.58%) than what was reported for CD-1 mice housed in barren cages in some studies (from 22.67 ± 6.6% to 40.33 ± 8.6%) [4,5], indicating some variability in stereotypy level between research institutions or breeding facilities.

### Effects of stereotypy expression on recurrent perseveration

We found no association between the expression of stereotypies in CD-1 mice and our primary or secondary measures of recurrent perseveration in the guessing task. These results are consistent with results by Gross et al. [4,5] and Latham and Mason [43], indicating that stereotypies in this strain may not reflect recurrent perseveration. We also found no association between stereotypy level and perseveration score, the primary measure of recurrent perseveration in the guessing task, in C57BL/6 mice. However in this strain, the level of RT, but not BM, was positively correlated with the number of repetitions, indicating that in RT mice the pattern of responding was less random.

Some other studies have similarly only found correlations between stereotypic behaviour and repetitions in guessing tasks, which were interpreted as perseverative responses [4,45]. Since mice with higher numbers of repetitions also made fewer correct (i.e. rewarded) choices, they may have been more perseverative (i.e. inappropriately repeating a response). Although a previous study found a clear positive correlation between perseveration score, which measures higher order patterns of non-random responding and stereotypy level in this strain [42], our results provide evidence that stereotypy level in C57BL/6 mice may reflect impaired behavioural inhibition. However, in contrast to Garner et al. [42] who found a correlation between overall stereotypy level and recurrent perseveration, the correlation with perseveration was restricted to RT in the present study. Clearly, more studies are needed using other strains of mice and other test paradigms to assess recurrent perseveration, before we can draw firm conclusions about the relation between stereotypic behaviour and impaired inhibitory control of behaviour in laboratory mice. However, similar to studies in primates [31] and mink [45], it seems that at least in C57BL/6 mice, this relation may be dependent on the form of stereotypy.

The absence of a clear relation between recurrent perseveration and stereotypies in mice and other species [35,45] makes drawing conclusions difficult. However, a possible reason for inconsistent results is the methods used to measure impaired behavioural control. Behavioural inflexibility and impaired inhibition may be a result of different central nervous system regions [59,60], which can manifest in different forms of behaviour, such as reversal learning [33,61], impulsive responding [35,62], set shifting [63] among others, which could differentially be related to different stereotypies.

### Effects of stereotypy expression on cognitive bias

Responses to different arms in the cognitive bias task were similar to other spatial cognitive bias tasks [64,65], with mice spending more time in near positive arms compared to near negative arms. The clear discrimination between ambiguous arms indicates that mice associated near positive arms with a positive outcome and near negative arms with a negative outcome. It is possible that this difference may merely be a consequence of the close proximity to the positive arms. However, as mice spent approximately 30% of their time in the central arena and did not simply transverse from one arm to another, this explanation is unlikely. Future studies could systematically evaluate this hypothesis by rearranging ambiguous arms so that they do not mirror each other, thereby eliminating any effects of spatial proximity.

Stereotypy expression affected exploration of the maze in both strains. In CD-1 mice, increasing preference for the reference arms and avoidance of ambiguous arms was associated with increasing levels of BF. This pattern of exploration is consistent with the one previously reported for stereotyping CD-1 mice in the same task [26] and could reflect a general avoidance of novel unfamiliar arms (neophobia) [66,67]. However, in a radial maze paradigm similar to the one used here, rats which had experienced removal of housing enrichment spent more time in positive reference arms and avoided novel ambiguous arms suggestive of impaired welfare [48]. An alternative explanation for avoidance of ambiguous arms would be that BF mice were behaviourally less flexible and less responsive to the changed environment when all arms were available to explore. However, as stereotypy level selectively affected time spent in ambiguous arms, but not the number of entries to these arms, these data suggest that reduced time spent in ambiguous arms reflects an increased expectation of an aversive outcome.

Taken together, we conclude that avoidance of ambiguous arms indicates that mice with high levels of BF were displaying a negative cognitive bias. However, this is in contrast with a previous study [25], where high stereotyping mice displayed a more positive cognitive bias. Although both of these conflicting findings are preliminary until replicated, there would be two possible explanations for this difference if both findings were true: they are based on different forms of stereotypy and on different task paradigms. That different stereotypies may differ in their relationship with measures of cognitive bias has been reported in non-human primates [22]. It is also one of the findings of the present study, although here, no opposite effect of other forms of stereotypy on cognitive bias was found. However, mice with higher levels of BF were more active and their activity did not decrease across training sessions. Anxiety usually increases when mice are exposed to a novel environment, but over time they habituate as they explore the environment which is associated with a decrease in activity [68,69]. High stereotyping animals are generally more active [1,5,11], but the lack of decrease in activity during training in BF mice could indicate sensitization to the apparatus and possibly higher levels of stress and anxiety in these mice. Indeed, chronic stress has been shown to induce hyper-locomotion in exploration based tests, which can be triggered by acute stressors such as bright light [70]. Thus, the cognitive bias measured in BF mice could reflect increased anxiety levels.

An alternate explanation for these conflicting results is related to the task paradigm and the outcome measures used. Stereotyping animals are generally quicker to make a choice [11,45,71] and the positive bias found in the task based on differential food rewards reported by Novak et al. [25] may have been a result of more impulsive choices made to ambiguous cues and not related to affective valence. The relative difference between the positive and the negative outcome usually varies in cognitive bias tasks, and is likely a contributing factor to inconsistent results or difficulties in finding predicted cognitive biases [14,72,73]. Animals are less likely to anticipate the negative (or less positive) outcome in tasks using reward based outcomes [20,74,75]. Conversely, increasing the relative difference between the positive and the negative outcome, as used in the present study, may be better at detecting variation in expectations of a more negative outcome [14,73]. Although these differences require further investigation, they may be important in studying specific types of bias by choosing particular reinforcer values as certain types of cognitive bias may be more tightly linked to particular affective disorders. For example, differences in anticipation of positive events may be more relevant to depression-like states, whereas biases in anticipation of negative events have been linked to anxiety-like states (for review see Mendl et al. [14]).

Similar to BF mice in the CD-1 strain, C57BL/6 mice with higher stereotypy levels showed a lack of habituation and a higher preference for positive arms; however, unlike BF mice, C57BL/6 mice with higher levels of stereotypy did not exhibit a negative cognitive bias. Furthermore, regardless of whether performed alone or in combination with another stereotypy, BM had no effect on performance in the cognitive bias task. This parallels results reported by Novak et al. [25], using an active choice task to assess cognitive bias, where no evidence for cognitive bias was found in BM mice in both CD-1 and C57BL/6 mice. Development of BM in CD-1 mice has been linked to behavioural [3,7] and physiological [12] measures of stress; however, in adult mice it was found to vary strongly depending on circumstances [25,43]. Therefore it could be linked to a more general arousal or motivation to explore the outside environment [3,7] and therefore dissociated from affective states.

One of the limitations of the present study is that it only looked at stereotypies at one time point. Stereotypies develop gradually from source behaviours, generally increasing in frequency and duration while becoming less variable with time [3,76]. When fully established, stereotypies may become emancipated from the initial causal factors [77]. In CD-1 mice, it was found that stereotypies may not yet be fully established at 11 months of age [5], and the developmental stage of different stereotypy forms (and in individuals displaying the same form) may have varied in the present study. For example, in deer mice, jumping develops earlier and faster compared to back-flipping [78], possibly due to the greater complexity of back-flipping. Understanding the etiology of stereotypic behaviour, both between and within forms, and the casual factors which contribute to their development still remains an area where critical information is lacking.

Taken together, there was no consistent pattern of responses across strains of mice and forms of stereotypies that could explain the results of the present and previous studies on the relationship between stereotypic behaviour and cognitive bias in laboratory mice [25,26]. However, both cognitive bias test paradigms used so far have not been successfully validated. Therefore, further validation is needed before drawing firm conclusions about the relationship between stereotypic behaviour and cognitive biases in laboratory mice.

## Conclusions

While the cognitive bias paradigm used in this study needs further validation, our results mirror findings found in other species, where only some forms of stereotypies were linked to recurrent perseveration [31,45] or associated with cognitive bias, even if performed by the same individual [22]. Our findings therefore further support the idea that different forms of stereotypy should be considered separately in studies about their relationship with brain function, affective state or other measures of animal welfare. Besides the welfare implications this may have, our data also imply that the form and level of stereotypy performed may influence the outcome of behavioural studies using exploratory behaviour, novelty seeking and choice based tasks, and in turn may contribute to variability in the data and poor reproducibility between studies.

## Supporting Information

S1 Table Number of mice for each stereotypy at the time of screening, testing and used for analysis. 78 mice from each strain were screened for stereotypy forms, and 60 of each strain were chosen for testing.
(PDF)

S2 Table Measures of recurrent perseveration in the guessing task for each strain. Data are presented as mean ± SEM.
(PDF)

S3 Table Mean ± SEM of exploration measures in the radial maze during training and testing for each strain. (PDF)

## References

[1] Odberg FO. The jumping stereotypy in the bank vole (Clethrionomys glareolus). Biol Behav 1986;11:130–43.

[2] Mason GJ. Stereotypies?: a critical review. Anim Behav 1991;41:1015–37.

[3] Würbel H, Stauffacher M, von Holst D. Stereotypies in Laboratory Mice-Quantitative and Qualitative Description of the Ontogeny of "Wire-gnawing" and "Jumping " in Zur?: ICR and ZunICR nu. Ethology 1996;102:371–85.

[4] Gross AN, Engel AKJ, Richter SH, Garner JP, Würbel H. Cage-induced stereotypies in female ICR CD-1 mice do not correlate with recurrent perseveration. Behav Brain Res 2011;216:613–20. 10.1016/j.bbr.2010.09.003 20837068

[5] Gross AN, Richter SH, Engel AKJ, Würbel H. Cage-induced stereotypies, perseveration and the effects of environmental enrichment in laboratory mice. Behav Brain Res 2012;234:61–8. 10.1016/j.bbr.2012.06.007 22721674

[6] Powell SB, Newman HA, Pendergast JF, Lewis MH. A rodent model of spontaneous stereotypy: initial characterization of developmental, environmental, and neurobiological factors. Physiol Behav 1999;66:355–63. 10336165 10.1016/s0031-9384(98)00303-5

[7] Nevison CM, Hurst JL, Barnard CJ. Why do male ICR(CD-1) mice perform bar-related (stereotypic) behaviour? Behav Processes 1999;47:95–111. 10.1016/S0376-6357(99)00053-4 24896933

[8] Wiedenmayer C. Causation of the ontogenetic development of stereotypic digging in gerbils. Anim Behav 1997;53:461–70. 10.1006/anbe.1996.0296

[9] Cooper JJ, Nicol CJ. Stereotypic behaviour affects environmental preference in bank voles, Clethrionomys glareolus. Anim Behav 1991;41:971–7.

[10] Cabib S. The neurobiology of stereotypy II: The role of stress In: Mason G, Rushen J, editors. Stereotypic Anim. Behav.—Fundam. Appl. to Welf. 2nd Edition, CABI; 2006, p. 227–55.

[11] Garner JP, Mason GJ. Evidence for a relationship between cage stereotypies and behavioural disinhibition in laboratory rodents. Behav Brain Res 2002;136:83–92. 12385793 10.1016/s0166-4328(02)00111-0

[12] Würbel H, Stauffacher M. Physical condition at weaning affects exploratory behaviour and stereotypy development in laboratory mice. Behav Processes 1998;43:61–9. 24897641 10.1016/s0376-6357(97)00086-7

[13] Würbel H, Stauffacher M. Age and weight at weaning affect corticosterone level and development of stereotypies in ICR-mice. Anim Behav 1997;53:891–900.

[14] Mendl M, Burman OHP, Parker RM, Paul ES. Cognitive bias as an indicator of animal emotion and welfare: Emerging evidence and underlying mechanisms. Appl Anim Behav Sci 2009;118:161–81. 10.1016/j.applanim.2009.02.023

[15] Paul ES, Harding EJ, Mendl M. Measuring emotional processes in animals: the utility of a cognitive approach. Neurosci Biobehav Rev 2005;29:469–91. 10.1016/j.neubiorev.2005.01.002 15820551

[16] Bar-Haim Y, Lamy D, Pergamin L, Bakermans-Kranenburg MJ, van Ijzendoorn MH. Threat-related attentional bias in anxious and nonanxious individuals: A meta-analytic study. Psychol Bull 2007;133:1–24. 17201568 10.1037/0033-2909.133.1.1

[17] Eysenck MW, Mogg K, May J, Richards A, Mathews A. Bias in interpretation of ambiguous sentences related to threat in anxiety. J Abnorm Psychol 1991;100:144–50. 2040764 10.1037//0021-843x.100.2.144

[18] MacLeod AK, Byrne A. Anxiety, depression, and the anticipation of future positive and negative experiences. J Abnorm Psychol 1996;105:286–9. 8723011 10.1037//0021-843x.105.2.286

[19] Bateson M, Desire S, Gartside SE, Wright G. Agitated honeybees exhibit pessimistic cognitive biases. Curr Biol 2011;21:1070–3. 10.1016/j.cub.2011.05.017 21636277 PMC3158593

[20] Brydges NM, Leach M, Nicol K, Wright R, Bateson M. Environmental enrichment induces optimistic cognitive bias in rats. Anim Behav 2011;81:169–75. 10.1016/j.anbehav.2010.09.030

[21] Burman O, McGowan R, Mendl M, Norling Y, Paul E, Rehn T, et al Using judgement bias to measure positive affective state in dogs. Appl Anim Behav Sci 2011;132:160–8. 10.1016/j.applanim.2011.04.001

[22] Pomerantz O, Terkel J, Suomi SJ, Paukner A. Stereotypic head twirls, but not pacing, are related to a "pessimistic"-like judgment bias among captive tufted capuchins (Cebus apella). Anim Cogn 2012;15:689–98. 10.1007/s10071-012-0497-7 22526692 PMC3635140

[23] Brilot BO, Asher L, Bateson M. Stereotyping starlings are more "pessimistic". Anim Cogn 2010;13:721–31. 10.1007/s10071-010-0323-z 20464439

[24] Keen HA, Nelson OL, Robbins CT, Evans M, Shepherdson DJ, Newberry RC. Validation of a novel cognitive bias task based on difference in quantity of reinforcement for assessing environmental enrichment. Anim Cogn 2013;17:529–41. 10.1007/s10071-013-0684-1 24045850

[25] Novak J, Stojanovski K, Melotti L, Reichlin TS, Palme R, Würbel H. Effects of stereotypic behaviour and chronic mild stress on judgement bias in laboratory mice. Appl Anim Behav Sci 2016:162–72.

[26] Novak J, Bailoo JD, Melotti L, Rommen J, Würbel H. An Exploration Based Cognitive Bias Test for Mice: Effects of Handling Method and Stereotypic Behaviour. PLoS One 2015;10:e0130718 10.1371/journal.pone.0130718 26154309 PMC4496074

[27] Garner JP, Mason GJ, Smith R. Stereotypic route-tracing in experimentally caged songbirds correlates with general behavioural disinhibition. Anim Behav 2003;66:711–27. 10.1006/anbe.2002.2254

[28] Frith CD, Done DJ. Stereotyped responding by schizophrenic patients on a two-choice guessing task. Psychol Med 1983;13:779–86. 6665094 10.1017/s0033291700051485

[29] Vickery SS, Mason GJ. Stereotypy and perseverative responding in caged bears: further data and analyses. Appl Anim Behav Sci 2005;91:247–60. 10.1016/j.applanim.2005.01.005

[30] Hemmings A, McBride SD, Hale CE. Perseverative responding and the aetiology of equine oral stereotypy. Appl Anim Behav Sci 2007;104:143–50. 10.1016/j.applanim.2006.04.031

[31] Pomerantz O, Paukner A, Terkel J. Some stereotypic behaviors in rhesus macaques (Macaca mulatta) are correlated with both perseveration and the ability to cope with acute stressors. Behav Brain Res 2012;230:274–80. 10.1016/j.bbr.2012.02.019 22366267 PMC3635133

[32] Tanimura Y, Yang MC, Lewis MH. Procedural learning and cognitive flexibility in a mouse model of restricted, repetitive behaviour. Behav Brain Res 2008;189:250–6. 10.1016/j.bbr.2008.01.001 18272239

[33] Judge PG, Evans DW, Schroepfer KK, Gross AC. Perseveration on a reversal-learning task correlates with rates of self-directed behavior in nonhuman primates. Behav Brain Res 2011;222:57–65. 10.1016/j.bbr.2011.03.016 21419808

[34] Garner JP, Meehan CL, Mench JA. Stereotypies in caged parrots, schizophrenia and autism: evidence for a common mechanism. Behav Brain Res 2003;145:125–34. 10.1016/S0166-4328(03)00115-3 14529811

[35] Dallaire J, Meagher RK, Díez-León M, Garner JP, Mason GJ. Recurrent perseveration correlates with abnormal repetitive locomotion in adult mink but is not reduced by environmental enrichment. Behav Brain Res 2011;224:213–22. 10.1016/j.bbr.2011.03.061 21466825

[36] Groenewegen HJ. The basal ganglia and motor control. Neural Plast 2003;10:107–20. 10.1155/NP.2003.107 14640312 PMC2565420

[37] Odberg FO, Kennes D, De Rycke PH, Bouquet Y. The effect of interference in catecholamine biosynthesis on captivity-induced jumping stereotypy in bank voles (Clethrionomys glareolus). Arch Int Pharmacodyn Thérapie 1987;285:34–42. 3555375

[38] Frith CD. The cognitive neuropsychology of schizophrenia. Int J Psychol 2000;35:272–3. 10.1016/0191-8869(93)90350-C

[39] Sandson J, Albert ML. Varieties of perseveration. Neuropsychologia 1984;22:715–32. 10.1016/0028-3932(84)90098-8 6084826

[40] Frith CD, Done DJ. Stereotyped behaviour in madness and in health In: Cooper SJ, Dourish CT, editors. Neurobiol. stereotyped Behav., New York, NY, US: Clarendon Press/Oxford University Press; 1990, p. 232–59.

[41] Vickery S, Mason G. Behavioral persistence in captive bears: implications for reintroduction. Ursus 2003;1:35–43.

[42] Garner JP, Thogerson CM, Dufour BD, Würbel H, Murray JD, Mench JA. Reverse-translational biomarker validation of Abnormal Repetitive Behaviors in mice: an illustration of the 4P's modeling approach. Behav Brain Res 2011;219:189–96. 10.1016/j.bbr.2011.01.002 21219937 PMC3062660

[43] Latham N, Mason G. Frustration and perseveration in stereotypic captive animals: is a taste of enrichment worse than none at all? Behav Brain Res 2010;211:96–104. 10.1016/j.bbr.2010.03.018 20230861

[44] Feenders G, Bateson M. Hand rearing affects emotional responses but not basic cognitive performance in European starlings. Anim Behav 2013;86:127–38. 10.1016/j.anbehav.2013.05.002 23888084 PMC3719021

[45] Campbell DLM, Dallaire J, Mason GJ. Environmentally enriched rearing environments reduce repetitive perseveration in caged mink, but increase spontaneous alternation. Behav Brain Res 2012;239:177–87. 10.1016/j.bbr.2012.11.004 23159704

[46] Jones MA, Mason GJ, Pillay N. Correlates of birth origin effects on the development of stereotypic behaviour in striped mice, Rhabdomys. Anim Behav 2011;82:149–59. 10.1016/j.anbehav.2011.04.010

[47] Luria AR. Two kinds of motor perseveration in massive injury of the frontal lobes. Brain 1965;88:1–10. 10.1093/brain/88.1.1 14280275

[48] Franks B, Champagne F, Higgins ET. How enrichment affects exploration trade-offs in rats: implications for welfare and well-being. PLoS One 2013;8:e83578 10.1371/journal.pone.0083578 24376721 PMC3871681

[49] Gross AN, Engel AKJ, Würbel H. Simply a nest? Effects of different enrichments on stereotypic and anxiety-related behaviour in mice. Appl Anim Behav Sci 2011;134:239–45. 10.1016/j.applanim.2011.06.020

[50] Würbel H, Chapman R, Rutland C. Effect of feed and environmental enrichment on development of stereotypic wire-gnawing in laboratory mice. Appl Anim Behav Sci 1998;60:69–81. 10.1016/S0168-1591(98)00150-6

[51] Martin P, Bateson P. Measuring Behaviour: An Introductory Guide. 2nd edition Cambridge University Press; 1993.

[52] Lalonde R. The neurobiological basis of spontaneous alternation. Neurosci Biobehav Rev 2002;26:91–104. 11835987 10.1016/s0149-7634(01)00041-0

[53] Deacon RMJ, Rawlins JNP. T-maze alternation in the rodent. Nat Protoc 2006;1:7–12. 17406205 10.1038/nprot.2006.2

[54] Ridley RM. The psychology of perseverative and stereotyped behaviour. Prog Neurobiol 1994;44:221–31. 7831478 10.1016/0301-0082(94)90039-6

[55] Bailoo JD, Bohlen MO, Wahlsten D. The precision of video and photocell tracking systems and the elimination of tracking errors with infrared backlighting. J Neurosci Methods 2010;188:45–52. 10.1016/j.jneumeth.2010.01.035 20138914 PMC2847046

[56] Bates D, Maechler M, Bolker B, Walker S. lme4: Linear mixed-effects models using Eigen and S4. 2014. http://cran.r-project.org/web/packages/lme4/citation.html (accessed August 19, 2014).

[57] Akre AK, Bakken M, Hovland AL, Palme R, Mason G. Clustered environmental enrichments induce more aggression and stereotypic behaviour than do dispersed enrichments in female mice. Appl Anim Behav Sci 2011;131:145–52. 10.1016/j.applanim.2011.01.010

[58] Tilly S-LC, Dallaire J, Mason GJ. Middle-aged mice with enrichment-resistant stereotypic behaviour show reduced motivation for enrichment. Anim Behav 2010;80:363–73. 10.1016/j.anbehav.2010.06.008

[59] Annoni G, Pegna AJ, Michel CM, Estade M, Landis T. Motor Perseverations: A Function of the Side and the Site of a Cerebral Lesion. Eur Neurol 1998;14:84–90. 10.1159/000007963 9693237

[60] Gandola M, Toraldo A, Invernizzi P, Corrado L, Sberna M, Santilli I, et al How many forms of perseveration? Evidence from cancellation tasks in right hemisphere patients. Neuropsychologia 2013;51:2960–75. 10.1016/j.neuropsychologia.2013.10.023 24200919

[61] Izquierdo A, Wiedholz LM, Millstein RA, Yang RJ, Bussey TJ, Saksida LM, et al Genetic and dopaminergic modulation of reversal learning in a touchscreen-based operant procedure for mice. Behav Brain Res 2006;171:181–8. 10.1016/j.bbr.2006.03.029 16713639

[62] Agnoli L, Carli M. Dorsal-striatal 5-HT?A and 5-HT?C receptors control impulsivity and perseverative responding in the 5-choice serial reaction time task. Psychopharmacology (Berl) 2012;219:633–45. 10.1007/s00213-011-2581-0 22113450

[63] Garner JP, Thogerson CM, Würbel H, Murray JD, Mench JA. Animal neuropsychology: validation of the Intra-Dimensional Extra-Dimensional set shifting task for mice. Behav Brain Res 2006;173:53–61. 10.1016/j.bbr.2006.06.002 16842867

[64] Burman OHP, Parker RM, Paul ES, Mendl M. Anxiety-induced cognitive bias in non-human animals. Physiol Behav 2009;98:345–50. 10.1016/j.physbeh.2009.06.012 19560479

[65] Richter SH, Schick A, Hoyer C, Lankisch K, Gass P, Vollmayr B. A glass full of optimism: Enrichment effects on cognitive bias in a rat model of depression. Cogn Affect Behav Neurosci 2012;12:527–42. 10.3758/s13415-012-0101-2 22644760

[66] Griebel G, Belzung C, Misslin R, Vogel E. The free-exploratory paradigm: an effective method for measuring neophobic behaviour in mice and testing potential neophobia-reducing drugs. Behav Pharmacol 1993;4:637–44. 11224232

[67] Teixeira-Silva F, Antunes FD, Santos Silva PR, Goes TC, Dantas EC, Santiago MF, et al The free-exploratory paradigm as a model of trait anxiety in rats: Test-retest reliability. Physiol Behav 2009;96:729–34. 19385028 10.1016/j.physbeh.2009.01.008

[68] Lister RG. Ethologically-based animal models of anxiety disorders. Pharmacol Ther 1990;46:321–40. 10.1016/0163-7258(90)90021-S 2188266

[69] Crusio WE. Genetic dissection of mouse exploratory behaviour. Behav Brain Res 2001;125:127–32. 10.1016/S0166-4328(01)00280-7 11682103

[70] Strekalova T, Spanagel R, Dolgov O, Bartsch D. Stress-induced hyperlocomotion as a confounding factor in anxiety and depression models in mice. Behav Pharmacol 2005:171–80. http://journals.lww.com/behaviouralpharm/Abstract/2005/05000/Stress_induced_hyperlocomotion_as_a_confounding.6.aspx (accessed November 26, 2014). 15864072 10.1097/00008877-200505000-00006

[71] Ijichi CL, Collins LM, Elwood RW. Evidence for the role of personality in stereotypy predisposition. Anim Behav 2013;85:1–7. 10.1016/j.anbehav.2013.03.033

[72] Parker RMA, Paul ES, Burman OHP, Browne WJ, Mendl M. Housing conditions affect rat responses to two types of ambiguity in a reward-reward discrimination cognitive bias task. Behav Brain Res 2014;274:73–83. 10.1016/j.bbr.2014.07.048 25106739 PMC4199117

[73] Hales CA, Stuart SA, Anderson MH, Robinson ESJ. Modelling Cognitive Affective Biases in Major Depressive Disorder using Rodents. Br J Pharmacol 2014;44:4524–38. 10.1111/bph.12603 PMC4199314 24467454

[74] Chaby LE, Cavigelli S, White A, Wang K, Braithwaite V. Long-term changes in cognitive bias and coping response as a result of chronic unpredictable stress during adolescence. Front Hum Neurosci 2013;7:328 10.3389/fnhum.2013.00328 23847501 PMC3701140

[75] Anderson MH, Munafò MR, Robinson ESJ. Investigating the psychopharmacology of cognitive affective bias in rats using an affective tone discrimination task. Psychopharmacology (Berl) 2013;226:601–13. 10.1007/s00213-012-2932-5 23239131

[76] Würbel H. The Motivational Basis of Caged Rodents' Stereotypies In: Mason G, Rushen J, editors. Stereotypic Anim. Behav.—Fundam. Appl. to Welf. 2nd Edition, CABI; 2006.

[77] Mason GJ. Stereotypies and suffering. Behav Processes 1991;25:103–15. 10.1016/0376-6357(91)90013-P 24923970

[78] Powell SB, Newman HA, McDonald TA, Bugenhagen P, Lewis MH. Development of spontaneous stereotyped behavior in deer mice: effects of early and late exposure to a more complex environment. Dev Psychobiol 2000;37:100–8. 10954835

