# Supplementary material for: Effect of Cage-Induced Stereotypies on Measures of Affective State and Recurrent Perseveration in CD-1 and C57BL/6 Mice
Source: PLoS One. 2016 May 4;11(5):e0153203. doi: 10.1371/journal.pone.0153203 (PMC4856387; doi:10.1371/journal.pone.0153203)
Supplement: S1 Table — 78 mice from each strain were screened for stereotypy forms, and 60 of each strain were chosen for testing. (PDF) [file pone.0153203.s001.pdf]

S1 Table

	Stereotypy form					
**CD-1**	**NS**	**BM**	**CT-BM**	**CT**	**BF**	**CIR**
At the time of screening	20	27	11	2	12	6
At the time of testing	14	24	7	0	9	6
Used for analysis	8	20	7	0	9	0
**C57BL/6**	**NS**	**BM**	**RT-BM**	**RT**	**CIR**	
At the time of screening	20	7	25	25	1	
At the time of testing	3	4	46	7	0	
Used for analysis	0	0	40	0	0	

NS – no stereotypy,
BM – bar-mouthing,
RT – route-tracing,
BF – back-flipping,
CIR – circling,
CT-BM – cage top twirling and bar-mouthing,
RT-BM – route-tracing and bar-mouthing
