# Supplementary material for: Effect of Cage-Induced Stereotypies on Measures of Affective State and Recurrent Perseveration in CD-1 and C57BL/6 Mice
Source: PLoS One. 2016 May 4;11(5):e0153203. doi: 10.1371/journal.pone.0153203 (PMC4856387; doi:10.1371/journal.pone.0153203)
Supplement: S2 Table — Data are presented as mean ± SEM. (PDF) [file pone.0153203.s002.pdf]

S2 Table

	**CD-1**	**C57BL/6**
perseveration score	2 ± 1	3 ± 0.5
repetitions	13 ± 1	10 ±1
alternations	10 ± 1	10 ± 1
p making correct choice %	52 ± 1	53 ± 1
p displaying side bias %	48 ± 2	50 ± 2
