# Supplementary material for: Effect of Cage-Induced Stereotypies on Measures of Affective State and Recurrent Perseveration in CD-1 and C57BL/6 Mice
Source: PLoS One. 2016 May 4;11(5):e0153203. doi: 10.1371/journal.pone.0153203 (PMC4856387; doi:10.1371/journal.pone.0153203)
Supplement: S3 Table — (PDF) [file pone.0153203.s003.pdf]

S3 Table

	training	testing
**CD-1**		
time in positive arms %	40 ± 1	22 ± 1
time in negative arms %	24 ± 1	11 ± 1
positive arm score	0.24 ± 0.02	0.34 ± 0.05
time in reference arms %	-	34 ± 1
time in ambiguous arms %	-	33 ± 1
reference arm score	-	0.01 ± 0.03
time in near positive arms %	-	19 ± 0.2
time in near negative arms %	-	15 ± 0.1
ambiguous arm score	-	0.12 ± 0.03
number of arms entered	57 ± 1	73 ± 4
reference arms entered %	-	46 ± 1
ambiguous arms entered %	-	54 ± 1
**C57BL/6**		
time in positive arms %	36 ± 0.4	18 ± 1
time in negative arms %	30 ± 0.5	14 ± 1
positive arm score	0.09 ± 0.01	0.18 ± 0.04
time in reference arms %	-	33 ± 0.1
time in ambiguous arms %	-	41 ± 1
reference arm score	-	- 0.12 ± 0.02
time in near positive arms %	-	22 ± 1
time in near negative arms %	-	19 ± 1
ambiguous arm score	-	0.08 ± 0.02
number of arms entered	52 ± 1	59 ± 2
reference arms entered %	-	47 ± 1
ambiguous arms entered %	-	53 ± 1
